# First person – Ivana María Gómez and Maia Abigail Rodríguez

**DOI:** 10.1242/bio.046409

**Published:** 2019-08-15

## Abstract

First Person is a series of interviews with the first authors of a selection of papers published in Biology Open, helping early-career researchers promote themselves alongside their papers. Ivana María Gómez and Maia Abigail Rodríguez are co-first authors on ‘Inhalation of marijuana affects *Drosophila* heart function’, published in BIO. Ivana is a biochemistry student from the National University of La Plata, Argentina, in the lab of Paola Ferrero, investigating endocannabinoid system physiology. Maia is a studying biotechnology and molecular biology in the lab of Paola Ferrero at the UNNOBA Foundation, Argentina, investigating signaling pathways inside cells.


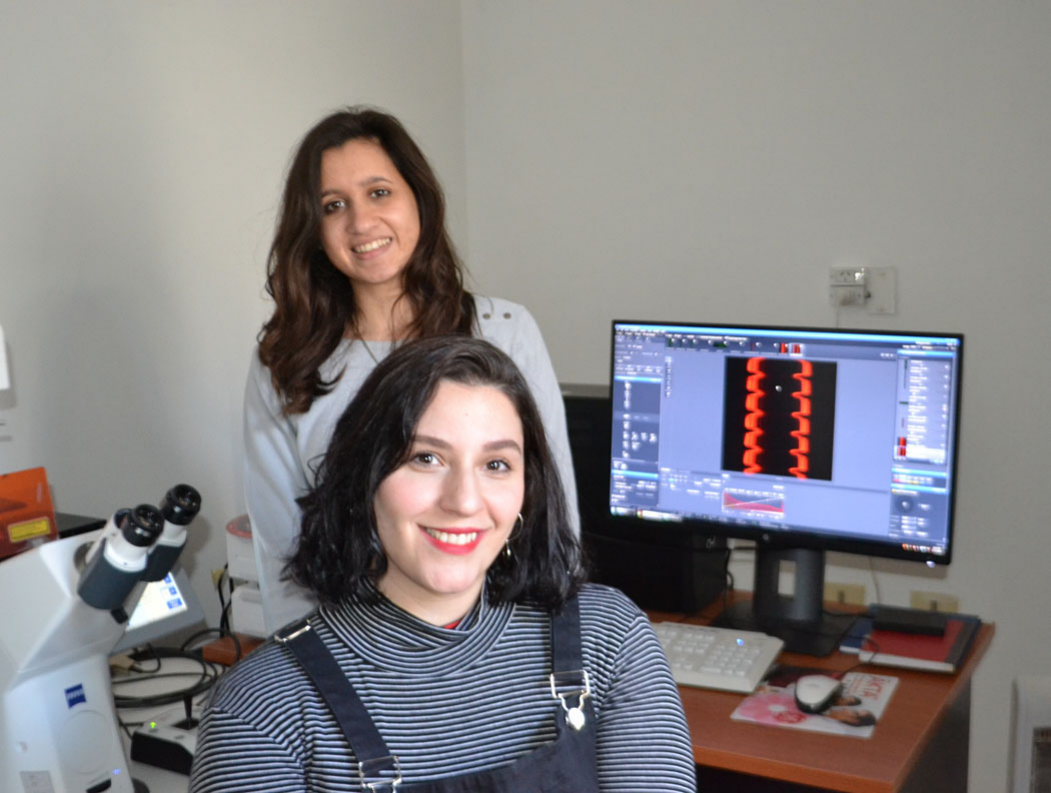


**Ivana María Gómez and Maia Abigail Rodríguez**

**What is your scientific background and the general focus of your lab?**

**I.M.G.**: I am a biochemistry student from the National University of La Plata, Argentina. In our country, to graduate in biochemistry, you must study for at least six years, and the curriculum itself comprises a wide range of subjects. Despite all the interesting subjects I have studied, I have always been interested in the physiology of organisms and in the pathophysiology of diseases, so over the last few years I have introduced myself to the physiology world. Three years ago, I began research in the medicinal cannabis field and I was fascinated by the complexity of the endocannabinoid system and the potential applications of its modulation. I have always known that I wanted to be a researcher, so as an undergraduate student, I joined Dr Paola Ferrero's research group, which studies cardiac physiology using the fruit fly as an animal model. It has been two years since I joined her group and everything we were working on is embodied in this paper. Thus, by fusing my scientific medicinal cannabis background and the laboratory's focus, we came to our current research topic: the impact of chronic cannabinoid treatment on the heart.

**M.A.R.**: I have a degree in biotechnology and molecular biology from the National University of La Plata. To obtain the degree you must complete a short thesis, so I decided to study the fruit fly because it seems to be a very interesting model with many advantages. So I joined the Cardiovascular Research Center ‘Dr Horacio E. Cingolani’ Group, led by Dr Ferrero. During my stay in her lab, I contributed to developing techniques that allowed us to obtain the first results of the phytocannabinoid's impact on the fly heart.

**How would you explain the main findings of your paper to non-scientific family and friends?**

In our article, we focused on finding out the effects produced by chronic consumption of a high-THC cannabis strain in *Drosophila melanogaster*’s heart. We administered two daily doses of cannabis to different groups of flies during two periods of time: a shorter and a longer treatment, and then we analyzed the heart function of each fly. In other experiments, we evaluated the survival of flies that were exposed to cannabis throughout their lives, to determine if this treatment affected their lifespan or not. Why did we use flies as a model? Because it has been proven that the main heart cells, called cardiomyocytes, are very similar between flies and mammals. Heart contraction and relaxation are controlled by changes in calcium concentration within the cardiomyocytes, and the proteins that are responsible for the regulation of this process are conserved between *Drosophila* and mammals; this allows us to utilize it as a model organism for cardiac physiology. Regarding the results obtained from the survival and mortality assays, we observed that the consumption of cannabinoids throughout life did not affect the lifespan of flies. In terms of cardiac function, the results can be divided into two groups: short-term and long-term effects. Short exposure to cannabis induced an increase in the arrhythmicity index, which means that the variability of heartbeat frequency increased. This was considered an early and transitory effect because it was not observed in individuals being exposed to the drug for a longer time. An early increment of the arrhythmicity index may be expected because heart performance should adapt to function in the presence of cannabis compounds. In addition to this, we evaluated heart contractility, which is the force the heart makes to contract, and there was a significant increase after cannabis inhalation for a prolonged period of time.

**What are the potential implications of these results for your field of research?**

Cannabis and cannabinoid therapies are a novel alternative to treat many diseases or clinical presentations that do not show favorable responses to conventional medications, such as refractory epilepsy, or pathologies that cause chronic pain; this is the case with fibromyalgia or arthritis, among others. However, a lot of research is still needed to provide information that ensures not only that these treatments are standardized, but also that the safety and efficacy conditions are appropriate. In this context, we believe that it is crucial to promote research in this field in order to better understand how cannabis' active compounds function in our body, and to be able to address the many questions that arise around its use in medicinal purposes. It is known that cannabinoids affect heart function, but the impact of prolonged consumption over time is not well described yet. Despite the fact that this research field is still taking its first steps, we believe that the data provided by our group can be used as a starting point for further investigations that could contribute to the knowledge on this subject and delve into the mechanisms that dominate the effects of cannabinoids in the heart. We do know there is a long way to go, however it is of great importance so that in the future we will not only be capable of supporting patients who might select this therapy, but we could also provide evidence of the effectiveness and safety of these treatments. Our results are the first evidence of the *in vivo* impact of cannabinoids in *Drosophila melanogaster.* The fruit fly is a useful, low-cost and high-performance model for future tests of cardiac phenotypes induced by cannabinoids. It provides a simple and affordable platform prior or complementary to mammalian models. This characterization of cardiac function under marijuana exposure opens new pathways for conducting genetic screenings using vaporized substances.

**Figure:**
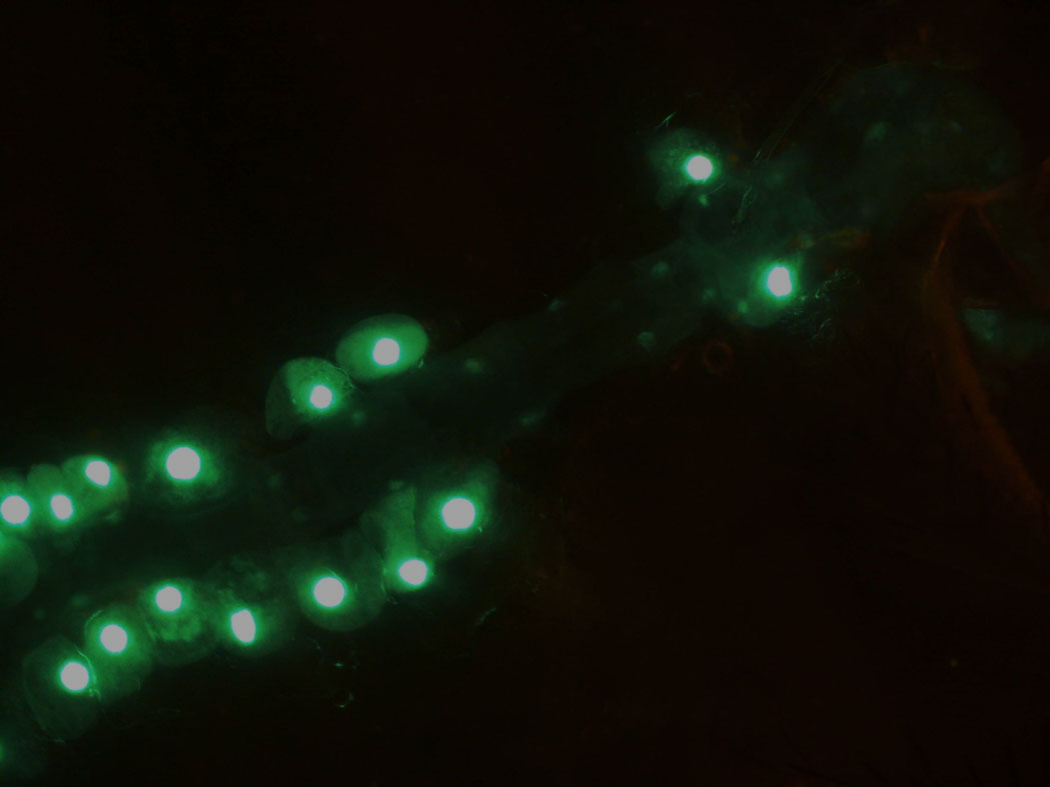
***Drosophila melanogaster*’s heart at 20× magnification, where the nuclei of cardiomyocytes and pericardial cells express green fluorescent protein (GFP), under the control of the Hand driver.**

**What has surprised you the most while conducting your research?**

In fact, almost everything was quite surprising; perhaps that is because we are just beginning our research careers. Many times it felt like some things were part of a science fiction movie, for example, the fact that a fly can ‘smoke’. It seemed unlikely that the heart of the fly and our own could work in such a similar way. The administration of cannabis to a group of flies and the observation of their hearts responding was a result itself, because there is no academic background for this kind of intervention in *Drosophila*, so we did not know what to expect from our experiments. It was a whole new world to explore! However, as we went deeper into this world, we started to become familiar with the experiments and results and we finally realized that what we are doing is not science fiction: it is science.

“In fact, almost everything was quite surprising; perhaps that is because we are just beginning our research careers.”

**What, in your opinion, are some of the greatest achievements in your field and how has this influenced your research?**

There are just a few research groups with experience in the pathophysiology in *Drosophila* of international scope; they have lain the foundations for using the *Drosophila* model to study aging and the effects of genetic abnormalities on cardiac function. Many of these genes are conserved between the fly and the human. In Argentina, Dr Paola Ferrero has set up her own research line based on her expertise on working with mammals and studies of proliferation and apoptosis in the *Drosophila* model. Taking into account the advantages of the model, she began to consolidate the line of research in the country, slowly, and even when the funding received was not enough to finance her work. The group has published in international journals, contributing to the development of cardiac pathophysiology field. These publications include studies on aging and the first characterization of the Bowditch effect (or staircase phenomenon), analyzing *Drosophila* mutants not yet explored in humans. Subsequently, the group consolidated the methodology for screenings of vaporized substances. In this context, studying the inhalation pathway of cannabinoids in *Drosophila* becomes more feasible than in mammals and, given its advantages, the fruit fly constitutes a platform for further analysis in other models. We have established collaborations with international research groups, well known to have an impressive trajectory in the study of the cardiac structure (Dr Achim Paululat and Heiko Harten, Osnabrück, Germany). These collaborations allowed us to access to the most advanced technology to perform the assays that we cannot carry out in our country, as well as being in touch with the scientific community in general.

**What changes do you think could improve the professional lives of early-career scientists?**

To carry out research in Argentina is not simple: our country has a history of successive economic crises, which makes the development of science and technology very difficult. Therefore, scientific activity and publications are carried out with limited resources. In fact, all the experiments that gave rise to our work were done without enough specific funding. The lack of economic resources also influences early-career scientists: very few of us can get scholarships that allow us to study for a PhD, and the opportunities we have to continue within the scientific system are very limited. Even with our limited work conditions, we appeal to our creativity and we put in a lot of effort to carry out the experiments to obtain quality results…and for sure, this is not the ideal situation for any scientist. There are a lot of capable and enthusiastic young scientists to do research, but we need to be able to access adequate equipment, reagents and possibilities to meet scientists from other countries to establish new collaborations between research groups, which could not only improve our investigations, but also contribute to our development as professionals and as people.

**What's next for you?**

**I.M.G.**: I am planning to graduate in the upcoming months. My goal is to keep doing research about the endocannabinoid system, but my near future depends on the possibility of a scholarship that allows me to study my PhD. If this happens, I would like to contribute to elucidating the mechanisms that govern the cardiac effects of cannabis use that we described in this work, as well as identify the receptor(s) that mediate these effects in the fruit fly, which do not present any of the canonical receptors found in mammals. Finally, I would like to highlight that I am planning to carry out the analysis of cardiac function in *Drosophila*’s model of human diseases, such as Parkinson's and epilepsy, which are known to present cardiovascular compromise. I want to study the effect of cannabinoids in the heart of these flies, as cannabinoids are a therapeutic alternative to test-treat these diseases.

**M.A.R.**: I am planning to keep doing research. I would like to start my PhD as soon as possible. I am going to change the research field to study intracellular calcium cycling in bovine sperm. Although it is a totally different topic, I have already gained experience in the use of confocal microscopy and intracellular calcium analysis during the development of the experiments for this paper, which interestingly will be particularly useful for my PhD studies.
